# Platelet HLA gene bank digital matching technology for platelet transfusion refractory patients with malignant tumors: a case report

**DOI:** 10.3389/fonc.2024.1419485

**Published:** 2024-09-26

**Authors:** Xiang Gao, Jianhua Li, Xiuwen Ni, Lijun Wang, Peilin Hu, Xiaowei Zhu

**Affiliations:** ^1^ Department of Blood Transfusion, Haining People’s Hospital, Haining, Zhejiang, China; ^2^ Laboratory Department, Jiaxing Central Blood Station, Haining, Zhejiang, China

**Keywords:** HLA, platelet, PTR, gene bank, malignant tumor

## Abstract

The platelet human leucocyte antigen (HLA) gene bank contains the genetic information of HLA loci in a large number of blood donors. Currently, the most effective treatment for platelet transfusion refractoriness (PTR) is to evaluate the probability of antigenic mismatch by HLA genotyping of patients and to select HLA-matched donors in the gene bank through an information system. This case report describes the treatment of PTR in patients with malignant tumors using the platelet HLA gene bank digital matching technique. The analysis of individual cases will help guide transfusion strategies for such patients.

## Introduction

Platelet transfusion refractoriness (PTR) refers to the absence of a significant increase in platelet (PLT) count and improvement in clinical bleeding symptoms after two or more consecutive transfusions of sufficient doses of platelets (1, 2). Platelet transfusion efficacy is usually assessed using the platelet corrected count increment (CCI) (3, 4). CCI was calculated using the following formula: PLT gain after infusion (×10^9^) × body surface area (m^2^)/PLT infused (10^11^), body surface area = 0.0061 × height (cm) + 0.0128 × weight (kg) − 0.01529.

The effective reference values for infusion were 1-h CCI ≥ 7.5 or 24-h CCI ≥ 4.5. The causes of PTR usually include non-immune and immune factors, which can be caused by one factor alone or a mixture of the two factors (1, 5, 6). Non-immune factors include infection, fever, persistent bleeding, splenomegaly or hypersplenism, disseminated intravascular coagulation, drug use, microthrombus, and platelet quality problems. Non-immune factors are present in 80%–90% of PTR patients. Immune factors include the presence of human leucocyte antigen (HLA) antibodies, human platelet antigen (HPA) antibodies, CD36 antibodies, ABO antibodies, autoantibodies, immune complexes, and drug antibodies. Studies have shown that approximately 70%–85% of immune PTR cases are caused by HLA class I antibodies and that approximately 15%–30% of cases are caused by HPA or CD36 antibodies (1, 6–8). HLA class I antigens can be expressed on the surface of platelets and a tissue antigen closely related to human immunity. These HLA class I antibody-mediated immune responses affect the effectiveness of platelet transfusion by triggering platelet destruction (7, 9, 10). It has been reported that 30% to 70% of patients with immune thrombocytopenia who experience multiple transfusions may develop PTR while receiving platelets from randomized donors (11, 12). The conventional treatment of immune PTR includes platelet transfusion and the use of recombinant human thrombopoietin, but their therapeutic effect is not always effective (13).

In recent years, with the continuous development of HLA genotyping technology, it has become possible to use “allele match” to replace “antigen match”. Due to the polymorphism of the HLA antigen system on individual platelets, in order to find donors based on gene digital matching, it is necessary to establish a gene database containing a sufficient number of platelet donors so that most patients can find suitable donors from a wide antigen group. The patients’ HLA-I antibodies and HLA class I genotyping were detected. According to the principle of donor-specific antibody avoidance and donor-recipient cross-reactive group (CREG) matching grade, the matched donors were searched digitally in the platelet gene database through the information system. To achieve rapid and accurate matching of suitable platelet donors, this strategy provides an effective solution to the problem of immune PTR. Zhejiang Provincial Blood Center has established a platelet HLA gene bank information system covering the whole province, including clinical application, laboratory testing, blood donation service, blood distribution, and other modules. As of the end of 2023, more than 17,000 voluntary donors with known HLA typing have been registered. The average time from applying for blood transfusion to obtaining platelets available for transfusion is 3 days. In case of emergency, the task can be distributed to the nearest sub-center to shorten the matching time.

## Case report

**Figure 1:**
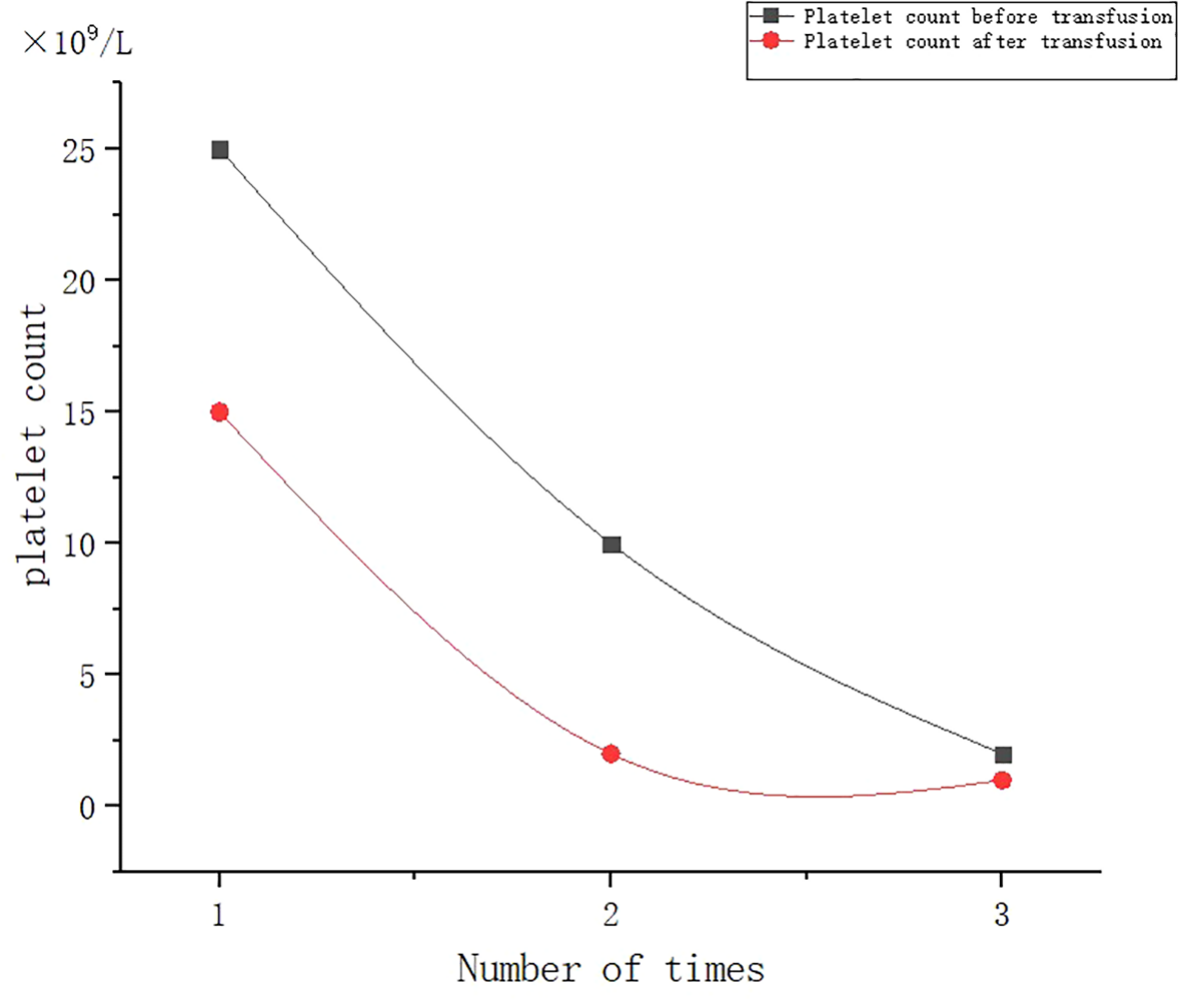
Effect of randomized donor platelet transfusion therapy when myelosuppression is induced after chemotherapy.

**Figure 2:**
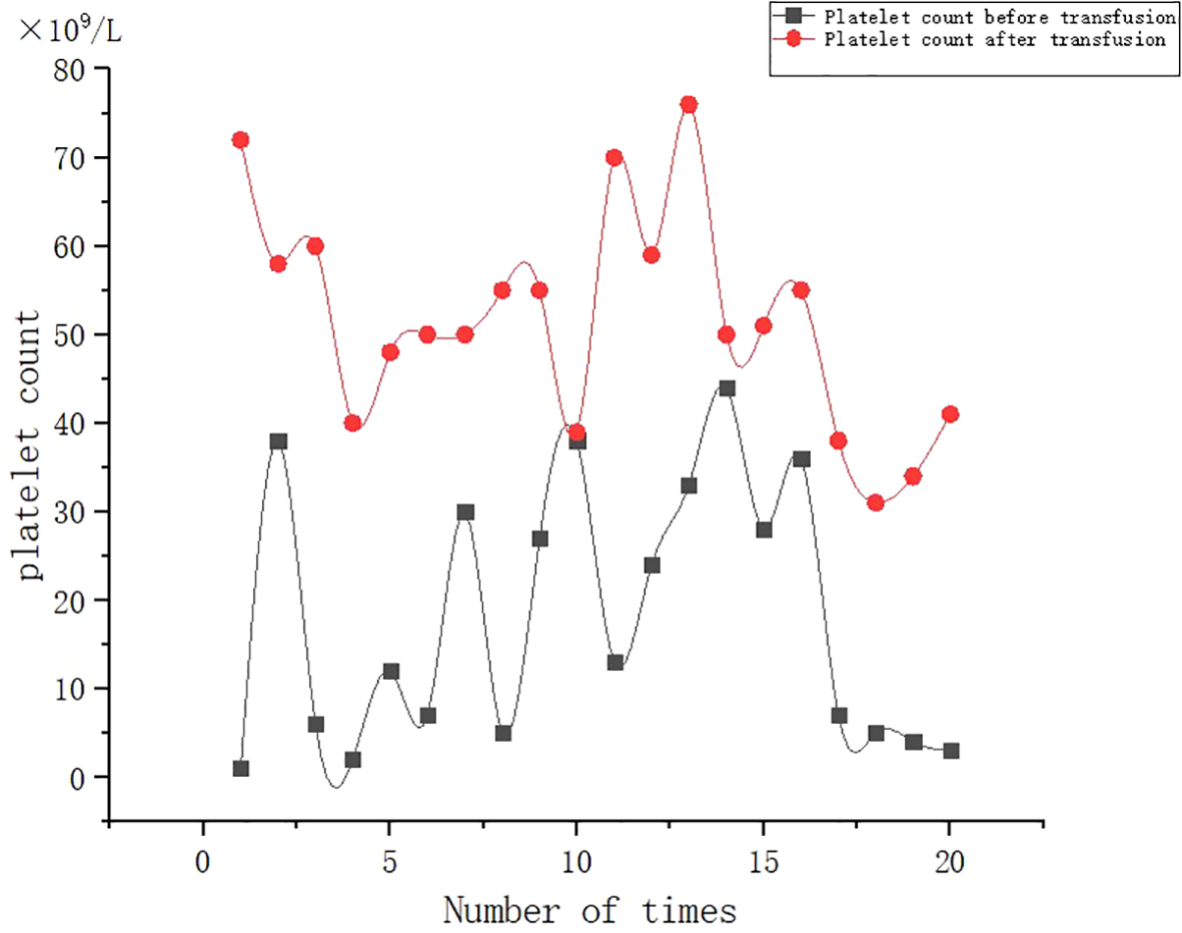
Effect of gene-matched platelet transfusion therapy.

**Figure 3:**
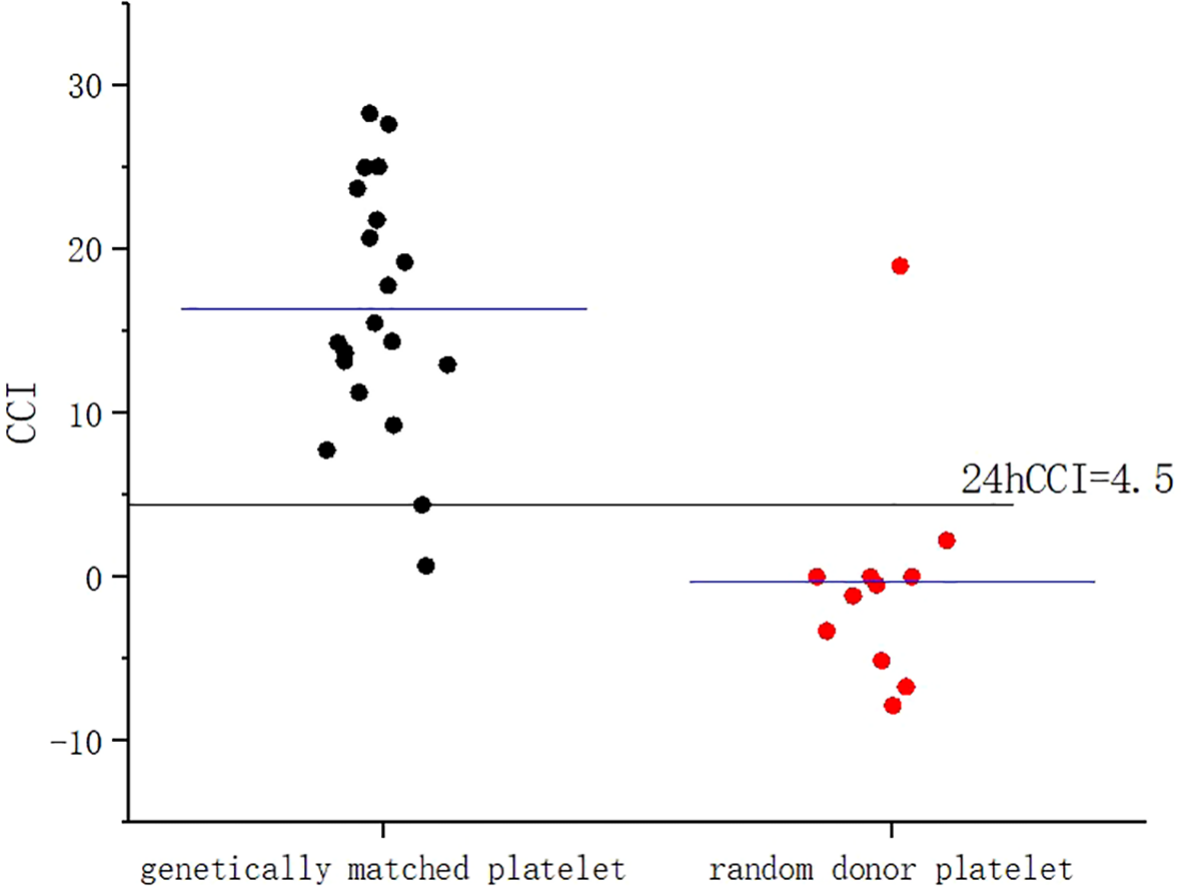
Comparison of 24-h CCI values between gene-matched platelets and random donor platelets. CCI, corrected count increment.

**Figure 4:**
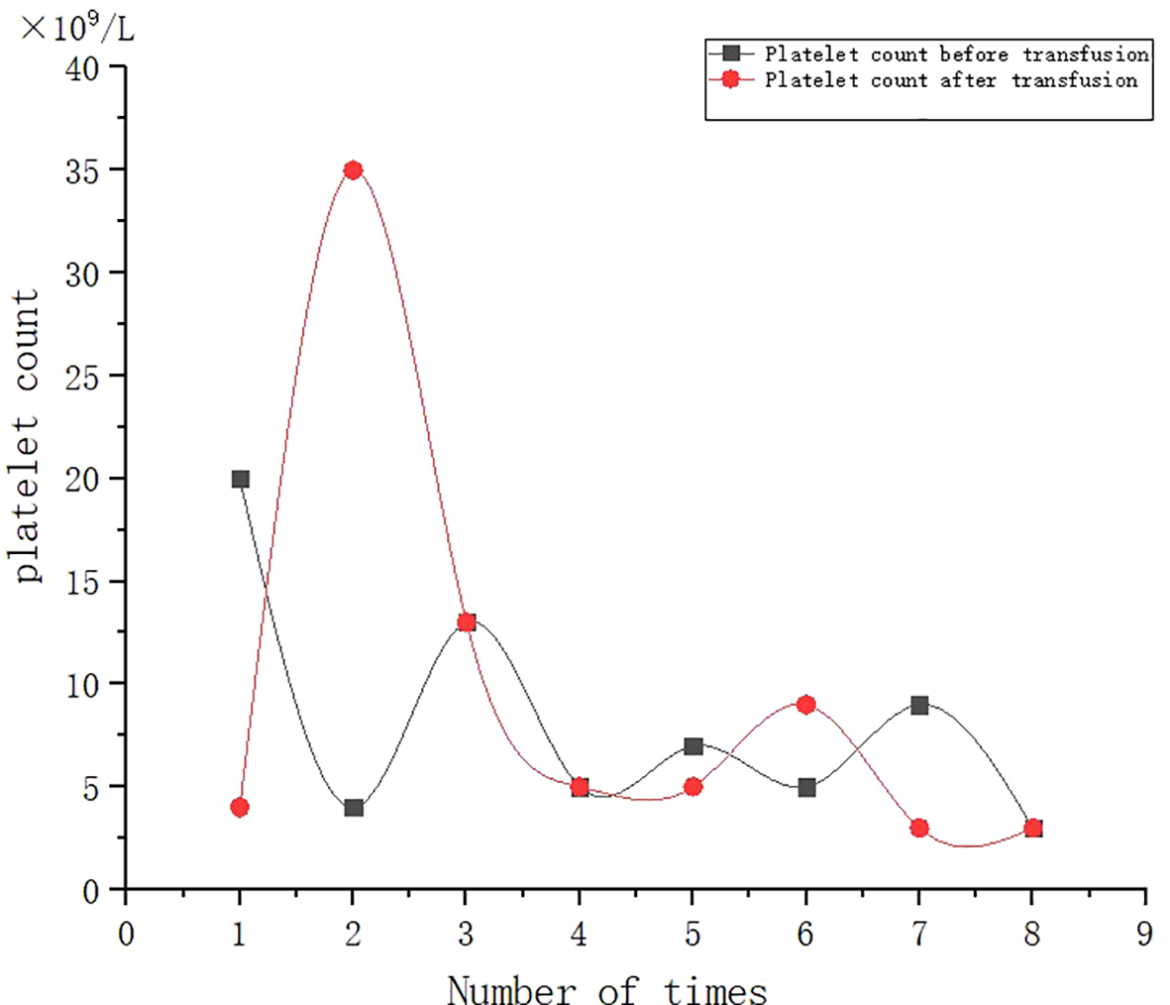
Effect of randomized donor platelet transfusion therapy.

A 53-year-old female patient was diagnosed with acute myeloid leukemia (M4) by bone marrow biopsy in September 2021. Bone marrow flow cytometry showed that blasts accounted for 36.8% and immature monocytes accounted for 9.6%. Leukemia fusion genes MLL-(AF6, ELL, ENL) were positive, and the rest of the genes were negative. Bone marrow staining showed a karyotype of 46, XX, t (6:11) (q27: q23) (15)/46, XX (5). Blood routine showed a white blood cell count of 5.4 × 10^9^/L, a hemoglobin concentration of 63 g/L, and a platelet count of 371 × 10^9^/L. The IA regimen was implemented in October 2021, which consisted of Zavedos 10 mg/day for three consecutive days followed by 2 days off and Cytosar 70 mg/day for one to seven consecutive days. The results of bone marrow reexamination after treatment showed that the proportion of blasts was 65%, the proportion of naive monocytes was 8.5%, and there was no sign of remission. Azacitidine combined with Venclexta has been used since December 8, 2021. During the third cycle of chemotherapy, the patient developed myelosuppression, manifested as fever and gastrointestinal bleeding. A blood routine examination showed a white blood cell count of 0.4 × 10^9^/L, a hemoglobin concentration of 81 g/L, and a platelet count of 48 × 10^9^/L. There was no response to recombinant human thrombopoietin. The platelet count did not increase after three consecutive infusions of 35 units of apheresis platelets from random donors (**Figure 1**). Forty-seven HLA-I class antibodies with mean fluorescence intensity (MFI) > 500 were detected by HLA antibody-specific test in the patient’s blood samples. The detection method was the panel reactive antibodies (PRA) magnetic bead method. MFI value classification was strongly positive, 14 kinds; positive, 7 kinds; weakly positive, 26 kinds (**Table 1**), which was consistent with HLA antibody-mediated immune PTR. HLA typing results were HLA-A*11:01:11:01; HLA-B*15:02:46:01. In the past, random donor platelets were selected and cross-matched with patient sera using serological methods such as solid-phase agglutination tests (14, 15). On the one hand, the number of platelets available for selection is limited, and the matching time is long. On the other hand, complex multi-specific and mixed antibodies are developed in many patients due to multiple transfusions, or antibodies against high-frequency antigens (such as HPA-1a, 4a, and CD36) make it difficult to find matched platelets by serological matching strategy. It is therefore rarely available. In this context, the choice of a rapid platelet screening method that can avoid immune rejection becomes critical. After evaluation, we used platelet HLA gene bank digital matching technology to find suitable platelet donors for patients. Based on the platelet gene bank digital matching information system, an automatic search was carried out according to the order of blood type, antigen, antibody avoidance, and CREG matching level. The patient’s entire HLA-1 antibody-positive list was first avoided. Then, based on HLA-I genotyping A and B loci, gene matching and CREG were performed. According to the international HLA matching classification standard, A, BU, and B2U were the best matching types; BX and BUX were the matching types crossed by one antigen, and B2X and BU2X were the matching types crossed by two antigens. Donors with high matching levels should be selected as much as possible. On January 25, 2022, 19 units of HLA gene-matched apheresis platelets were successfully transfused, and the matching grade was BUX. The 24-h CCI of this transfusion was 27.62, showing a significant increase in platelet count. During the whole treatment, the patient received a total of 20 gene-matched platelets, with CREG matching grades of 13 BUX and 7 BX. Among them, the effective infusion was 18 times, the average 24-h CCI value was 17.84, and two BX grade infusions were ineffective (24-h CCI < 4.5) (**Figures 2**, **3**, **Table 2**). During this time, the patient also received random donor platelets 11 times because the database was not always matched to a suitable donor in a timely way. The results showed that only one infusion achieved an effective effect (24-h CCI = 18.95), and the remaining 10 infusions failed to increase the platelet value or 24-h CCI < 4.5 (**Figures 3**, **4**).

**Table 1:** List of positive HLA antibodies in patient blood samples.

HLA antibody test results			
Antibody results	Specificity	MFI	Molecular specificity
Strongly positive	B81	14,662.48	B*81:01
	A66	13,216.66	A*66:01
	B45	13,063.41	B*45:01
	B48	12,205.34	B*48:01
	B50	12,001.25	B*50:01
	B44	11,722.03	B*44:01
	B73	11,497.90	B*73:01
	B61	11,460.40	B*40:01
	B27	11,390.97	B*27:01
	B47	11,234.79	B*47:01
	B41	10,691.96	B*41:01
	Cw2	10,662.43	C*02:02
	B44	10,469.41	B*44:01
	B49	10,391.28	B*49:01
Positive	B13	9,809.05	B*13:01
	B60	9,469.51	B*40:01
	B61	8,974.15	B*40:02
	B27	8,348.83	B*27:08
	B7	7,620.61	B*07:02
	B13	7,293.63	B*13:02
	B67	6,626.51	B*67:01
Weakly positive	B39	4,936.08	B*39:01
	Cw15	4,778.15	B*15:02
	B42	4,770.01	B*42:01
	Cw5	4,408.47	C*05:01
	Cw18	3,818.89	C*18:02
	Cw6	3,751.41	C*06:02
	B55	3,311.62	B*55:01
	B76	2,843.83	B*15:12
	A32	2,786.61	A*32:01
	Cw17	2,289.97	C*17:01
	B78	1,837.38	B*78:01
	A29	1,781.23	A*29:02
	B38	1,567.14	B*38:01
	B51	1,299.18	B*51:01
	B54	1,111.60	B*54:01
	A29	1,092.82	A*29:01
	B72	849.14	B*15:03
	B82	838.04	B*82:01
	B52	826.87	B*52:01
	B56	819.07	B*56:01
	B59	775.71	B*59:01
	B8	770.07	B*08:01
	B37	736.21	B*37:01
	B51	714.54	B*51:02
	B18	602.18	B*18:01
	Cw4	599.79	C*04:01

HLA, human leucocyte antigen.

**Table 2:** CREG match grades and 24-h CCI values (platelet count × 10^9^/L).

Number	Grade	Pre-transfusion	Post-transfusion	24-h CCI
1	BUX	1	72	27.62
2	BUX	38	58	9.25
3	BX	6	60	24.98
4	BUX	2	40	28.28
5	BX	12	48	23.7
6	BUX	7	50	21.77
7	BUX	30	50	13.17
8	BUX	5	55	20.67
9	BX	27	55	13.69
10	BX	38	39	0.66
11	BUX	13	70	25.02
12	BUX	24	59	19.2
13	BUX	33	76	17.78
14	BX	44	50	4.39
15	BUX	28	51	11.25
16	BUX	36	55	7.74
17	BX	7	38	12.94
18	BUX	5	31	14.36
19	BUX	4	34	14.27
20	BX	3	41	15.49

CCI, corrected count increment.

## Discussion

In recent years, platelet applications based on gene matching have been reported in the literature (16–19). However, due to the limitations of the cost of database construction and the level of information construction, this technology has not yet been applied on a large scale, and its effectiveness has not been fully demonstrated. We believe that this case provides a valuable piece of information, in which we can obtain 20 gene-matched platelets and collect 11 random donor platelets as controls at the same time, which is not available in most reports, and fully demonstrates the effectiveness of gene-matched platelet transfusion. Serological cross-matching platelet transfusion is an effective method for the treatment of immune PTR. However, cross-matched platelets are more costly and require a longer waiting period than random donor platelets. In contrast, the digital platelet matching gene bank is an efficient and accurate blood transfusion system engineering. Through the HLA genotyping and antibody detection of patients, the gene bank search or targeted recruitment of blood donors in the physical blood bank immediately can achieve the precise matching of alleles, antigens, or epitopes so as to greatly shorten the matching time. Especially in this case of a PTR patient with bone marrow suppression caused by maintenance chemotherapy for a malignant tumor, the hematopoietic function of bone marrow was inhibited, and the platelet count decreased rapidly with the risk of bleeding. Therefore, rapid and effective platelet transfusion became an important treatment strategy. Platelet digital matching in the HLA gene bank can ensure that patients can obtain timely and appropriate platelet transfusion when needed, which is impossible to achieve by traditional manual cross-matching.

## Conclusion

The analysis of the case will help to enrich the research on platelet transfusion strategy in patients with myelosuppression and PTR and help to demonstrate the effectiveness of gene-matched platelet transfusion.

## Data Availability

The original contributions presented in the study are included in the article/supplementary material. Further inquiries can be directed to the corresponding author.

## References

[1] Hod E Schwartz J. Platelet transfusion refractoriness. Br J Haematol. (2008) 142(3):348–60. doi: 10.1111/j.1365-2141.2008.07189.x 18510692

[2] Cohn CS. Platelet transfusion refractoriness: how do I diagnose and manage? Hematol 2014 Am Soc Hematol Educ Program Book. (2020) 2020(1):527–32. doi: 10.1182/hematology.2020000137 PMC7727584 33275694

[3] Zhang JC Ni LH Tu Y Hu HX. Related donor platelet transfusion improves platelet transfusion refractoriness in hematological patients. Front Med. (2023) 10:983644. doi: 10.3389/fmed.2023.983644 PMC10014593 36936203

[4] Hill-Strathy M Pinkerton PH Thompson TA Wendt A Collins A Cohen R. Evaluating the appropriateness of platelet transfusions compared with evidence-based platelet guidelines: An audit of platelet transfusions at 57 hospitals. Transfusion. (2021) 61(1):57–71. doi: 10.1111/trf.16134 33078852

[5] Belizaire R Makar RS. Non-alloimmune mechanisms of thrombocytopenia and refractoriness to platelet transfusion. Transfus Med Rev. (2020) 34(4):242–9. doi: 10.1016/j.tmrv.2020.09.002 PMC7494440 33129606

[6] Chen X Zhao Y Lv Y Xie J. Immunological platelet transfusion refractoriness: current insights from mechanisms to therapeutics. Platelets. (2024) 35(1):2306983. doi: 10.1080/09537104.2024.2306983 38314765

[7] Kekomäki S Volin L Koistinen P Koivunen E Koskimies S Ruutu T. Successful treatment of platelet transfusion refractoriness: the use of platelet transfusions matched for both human leucocyte antigens (HLA) and human platelet alloantigens (HPA) in alloimmunized patients with leukaemia. Eur J Haematol. (1998) 60(2):112–8. doi: 10.1111/j.1600-0609.1998.tb01007.x 9508352

[8] Ma C Wang J Yang L Feng Y Fu L Guan X. A single-center investigational study of CD36 antigen deficiency and platelet alloantibody distribution in different populations in Northern China as well as platelet alloantibodies effect on pregnancy. Clinica Chimica Acta. (2019) 498:68–75. doi: 10.1016/j.cca.2019.08.009 31421121

[9] Juskewitch JE Norgan AP De Goey SR Duellman PM Wakefield LL Gandhi MJ. How do I … manage the platelet transfusion–refractory patient? Transfusion. (2017) 57(12):2828–35. doi: 10.1111/trf.14316 28960321

[10] Blandin L Dougé A Fayard A Bay JO Berlie G Pereira B. Platelet transfusion refractoriness and anti-HLA immunization. Transfusion. (2021) 61(6):1700–4. doi: 10.1111/trf.16358 33709433

[11] Karlström C Linjama T Edgren G Lauronen J Wikman A Höglund P. HLA-selected platelets for platelet refractory patients with HLA antibodies: a single-center experience. Transfusion. (2019) 59(3):945–52. doi: 10.1111/trf.15108 30575964

[12] Zavyalova D Abraha J Rao P Morris GP. Incidence and impact of allele-specific anti-HLA antibodies and high-resolution HLA genotyping on assessing immunologic compatibility. Hum Immunol. (2021) 82(3):147–54. doi: 10.1016/j.humimm.2021.01.002 33478842

[13] Liu X Liang X Liang J Li Y Wang J. Immune thrombocytopenia induced by immune checkpoint inhibitors in solid cancer: case report and literature review. Front Oncol. (2020) 10:530478. doi: 10.3389/fonc.2020.530478 33365266 PMC7750527

[14] Chavan A Sharma RR Saikia B Malhotra P. Efficacy of cross-match compatible platelets in multi transfused haemato-oncology patients refractory to platelet transfusion. Transfus Apheresis Sci. (2019) 58(6):102657. doi: 10.1016/j.transci.2019.09.010 31706911

[15] Chapman JM Linder W Knudson CM. Comparison of platelet antibody screen, crossmatching and HLA antibody testing in patients refractory to platelet transfusions. Transfus Apheresis Sci. (2023) 62(3):103622. doi: 10.1016/j.transci.2022.103622 PMC10256835 36535829

[16] Kreuger AL Haasnoot GW Somers JAE Tomson B van der Bon JG van Kraaij MGJ. Ensuring HLA-matched platelet support requires an ethnic diverse donor population. Transfusion. (2020) 60(5):940–6. doi: 10.1111/trf.15728 PMC7317777 32086954

[17] Seike K Fujii N Asano N Ohkuma S Hirata Y Fujii K. Efficacy of HLA virtual cross-matched platelet transfusions for platelet transfusion refractoriness in hematopoietic stem cell transplantation. Transfusion. (2020) 60(3):473–8. doi: 10.1111/trf.15664 31970799

[18] Juskewitch JE Gandhi MJ Kreuter JD Norgan AP. Development and performance characteristics of Platelet Virtual Crossmatch (PLT VXM), a software application for the evaluation and management of platelet transfusion–refractory patients. Transfusion. (2020) 60(10):2284–93. doi: 10.1111/trf.16025 32827167

[19] Xu D Sun C Yu L He Y Deng G Zhang J. Methodological study on the establishment of HLA/HPA gene bank of platelet donors and its clinical application. Indian J Hematol Blood Transfus. (2023) 39(1):123–31. doi: 10.1007/s12288-022-01547-9 PMC9135102 35669352

